# Progress in the study of nutritional status and selenium in dialysis patients

**DOI:** 10.1080/07853890.2023.2197296

**Published:** 2023-04-10

**Authors:** Meiran Cao, Shuai Zheng, Wenhua Zhang, Guicai Hu

**Affiliations:** a Department of Nephrology, Affiliated Hospital of Chengde Medical University, Chengde, China; b Department of Gastrointestinal Surgery, Affiliated Hospital of Chengde Medical University, Chengde, China

**Keywords:** Dialysis, selenium, malnutrition, chronic kidney disease

## Abstract

Malnutrition is very common in patients with chronic kidney disease, especially in those on maintenance dialysis. Malnutrition is one of the major factors affecting survival and death of dialysis patients, and reducing their activity tolerance and immunity. There are numerous and interacting risk factors for malnutrition, such as reduced nutritional intake, increased energy expenditure, hormonal disorders, and inflammation. Selenium, in the form of selenoproteins, is involved in many physiological processes in the body and plays an important role in maintaining redox homeostasis. Oxidative stress and infection are very common in dialysis patients, and selenium levels in dialysis patients are significantly lower than those in the healthy population. It has been shown that there is a correlation between selenium levels in hemodialysis patients and their nutrition-related indicators, and that selenium supplementation may improve malnutrition in patients. However, further studies are needed to support this conclusion and there is a lack of basic research to further characterize the potential mechanisms by which selenium may improve malnutrition in dialysis patients. The purpose of this review is to provide a comprehensive overview of factors associated with malnutrition in dialysis patients and to describe the progress of research on nutritional status and selenium levels in dialysis patients.

## KEY MESSAGES

- Malnutrition is very common and one of the main factors affecting the survival and mortality of dialysis patients
- Risk factors for malnutrition in dialysis patients are numerous and interact with each other; controlling and reducing these risk factors is important to improve the nutritional status of patients.
- The trace element selenium acts to improve the nutritional status of patients by reducing oxidative stress and inflammation in their bodies.

## 1. Introduction

The prevention and treatment of chronic kidney disease (CKD) has become an important public health concern globally, with about 10% adults worldwide having CKD and about 1.2 million people dying from CKD each year. CKD is expected to be the fifth leading cause of death worldwide by 2040 [1]. With the progress of CKD, the renal and glomerular filtration function of patients is gradually reduced, and toxins in the body are continuously accumulated. Renal failure and multiple system damage such as nerve, muscle, and respiratory and metabolic disorders occur towards the end stage of the disease. Renal replacement therapy should be done on time. It has been shown that 77.5% patients with end-stage renal disease (ESRD) have received renal replacement therapy (RRT), of which 43.1% were treated with dialysis [2].

Long-term dialysis can remove metabolic waste and excess water from the body of CKD patients; however, a series of complications such as malnutrition, disorders of calcium and phosphorus metabolism, and electrolyte imbalance can occur. The global prevalence rates of malnutrition in patients with stage 3–5 CKD who did not receive dialysis and those who needed to maintain dialysis were 11–54% and 28–54%, respectively [3]. It is evident that malnutrition is more common in maintenance dialysis patients. Malnutrition can lead to reduced immune function and physical activity in patients, is closely associated with various infectious and non-infectious complications, severely reduces patients’ quality of life, and is one of the main factors affecting the survival and mortality of dialysis patients [4–6].

Selenium (Se) is an essential trace element that inhibits oxidation and suppresses inflammation [7]. In recent years, scholars across the globe have conducted an increasing number of studies on the relationship between Se and chronic kidney disease and found a correlation between Se and malnutrition in dialysis patients, which has great potential research value. The purpose of this review is to provide a comprehensive compilation of factors associated with malnutrition in dialysis patients and assess the progress of research regarding the effects of Se on the nutritional status of dialysis patients.

## 2. Definition of malnutrition

Along with clinicians’ attention to malnutrition in dialysis patients and numerous scholarly studies on the etiology of malnutrition, a variety of terms, such as cachexia, malnutrition, protein-energy wasting(PEW, and malnutrition-inflammatory-atherosclerosis syndrome (MIA) have evolved to describe the malnutrition status of dialysis patients [8–11]. Although the general meanings of these terms are similar, there are subtle differences [12].

The International Society of Renal Nutrition and Metabolism (ISRNM) (2008) states that the term cachexia is not used to describe the malnutrition status of all dialysis patients; it is more often used to describe the final stage of malnutrition in dialysis patients. In contrast, the definition of malnutrition is intuitive and clear. It mainly emphasizes on muscle wasting, hypoalbuminemia, and other low nutritional markers in dialysis patients due to inadequate nutritional intake or excessive protein loss [13], and does not comprehensively describe other aspects of malnutrition in dialysis patients. With a comprehensive understanding of malnutrition, the relationship between malnutrition and inflammation and its comorbidities has come into focus, resulting in a new definition of malnutrition due to inflammation and comorbidities – ‘malnutrition-inflammation-atherosclerosis syndrome’ – that focuses more on the study of cardiovascular complications in patients with ESRD. With progress in research, scholars have found that malnutrition in dialysis patients not only stems from inadequate nutrient intake, but metabolism-related factors also play a large role in it. Therefore, in 2008, the ISRNM introduced the term ‘protein-energy wasting’ to highlight the complexity of malnutrition in dialysis patients and defined the term as a state of decreased protein and energy reserves in the body [14]. This definition is currently the most widely used. However, it has a shortcoming. The definition only considers albumin and cholesterol; therefore, it may cause some bias in assessment of the nutritional status of hyperlipidemic patients. Although body mass index (BMI) is included in the diagnostic criteria of this definition, obesity sarcopenia may still be overlooked [12].

These ‘definitions’ suggest that the etiology of malnutrition in dialysis patients is complex and that there is no uniformity in the diagnosis of malnutrition due to dialysis.

## 3. Related index of malnutrition in dialysis patients

At present, there are many indexes for evaluating malnutrition in patients. Subjective Global Assessment (SGA), Malnutrition-Inflammation Score (MIS), Geriatric Nutritional Risk Index (GNRI), Mini Nutritional Assessment (MNA), and Nutritional Risk Screening 2002 (NRS-2002) are widely used for dialysis patients.

In 1987, Detsky et al. [15] proposed SGA in order to evaluate the nutritional status of patients undergoing gastrointestinal surgery. SGA mainly evaluates the nutritional status of patients from six aspects (weight change, dietary intake, gastrointestinal symptoms, functional ability, complications, and physical examination). As SGA is simple, convenient, cost effective, and has been validated in patients with ESRD, Kidney Disease Outcomes Quality Initiative (K DOQI) recommends SGA as a routine tool for assessing the nutritional status of dialysis patients [16]. SGA can not only assess the nutritional status of patients, but has also been found to be associated with a high risk of nutrition-related death in dialysis patients [17–20]. In addition, in a meta-analysis, Khor et al. demonstrated that SGA is an effective tool for assessing the nutritional status of patients with acute kidney injury (AKI) [20]. However, SGA also has some limitations: it is highly subjective and its scoring results are highly variable among each rater, which means that SGA may not be able to accurately determine the degree of malnutrition in patients, and is more suitable for distinguishing patients with severe malnutrition and good nutrition. Therefore, in order to evaluate the accuracy of SGA, it is better to conduct a formal training for raters [21–24].

With an increasing emphasis on the role of inflammation in malnutrition in dialysis patients, MIS is derived from SGA and includes three new parameters: BMI, serum albumin level, and total iron binding capacity, which makes it a more comprehensive evaluation system than SGA [25–27]. Similar to SGA, MIS is significantly associated with mortality in dialysis patients [28–30]. However, MIS has the following advantages over SGA: it is associated with a higher risk of hospitalization in dialysis patients [28], inflammation and quality of life [31], and can predict the occurrence of cardiovascular events in dialysis patients [32]. It is an important tool for identification of early malnutrition in patients with renal failure [6]. Dialysis-malnutrition score (DMS) [33,34], another SGA-based nutritional assessment tool, can also assess the nutritional status and cardiovascular disease risk in dialysis patients.

Both SGA and MIS are subjective. In order to seek a simpler and more objective nutritional assessment method, Bouillanne et al. [35] invented GNRI in 2005, which includes only three objective parameters: weight, height, and serum albumin level. The calculation formula is as follows: GNRI = [14.89 * albumin g/dL] + [41.7 + (weight/ideal weight)], < 82, major nutrition-related risk; between 82 and < 92, moderate nutrition-related risk; between 92 and ≤ 98, low nutrition-related risk; > 98, no risk. GNRI can assess the nutritional status of dialysis patients [34,36], and the changes of its parameters are significantly correlated with all-cause mortality [37–39] and cardiovascular events [37,40]. In addition, low GNRI score is associated with an increased risk of CKD progression to ESRD [41]. Two recent studies have shown that simultaneous assessment of the patient ‘s GNRI and modified creatinine index (mCI) can stratify the risk and improve the predictability of mortality in dialysis patients [42,43]. GNRI can also evaluate the muscle strength of dialysis patients and is a useful tool for screening sarcopenia in dialysis patients [44,45]. Therefore, GNRI is a simple and effective nutritional assessment method for long-term dialysis patients.

MNA was originally used to screen the nutritional status of individuals over 65 years of age, and later proved to be equally applicable in the hemodialytic population [46]. Another study by Tsai et al. showed that MNA was more effective than SGA in predicting the risk of malnutrition in peritoneal dialysis patients [47]. NRS-2002 is the preferred nutritional risk screening tool for inpatients recommended by ESPEN [48]. A retrospective study by Li et al. [49] showed that NRS-2002 score was strongly associated with AKI risk; the risk of death in patients with low NRS-2002 score was significantly lower than that in patients with high NRS-2002 score in terms of short- and long-term survival. The original Nutritional Risk Index (NRI) [50] was created by Buzby et al. to assess the nutritional status of postoperative patients. In 2019, Japanese scholars described a new NRI (the Nutritional Risk Index-Japanese Hemodialysis (NRI-JH)) based on the characteristics of dialysis patients in Japan. The new index included only four objective indicators, namely BMI, serum albumin, and total cholesterol and creatinine levels [51]. Subsequent studies have confirmed [24,52] that NRI-JH is a useful tool for assessing the nutritional status of dialysis patients and that its score is significantly associated with sarcopenia in dialysis patients.

Bioelectrical impedance analysis (BIA) is also a nutritional assessment method that analyzes body composition [53]. It is a non-invasive tool based on the conduction of human AC current to estimate the hydration state of human body [54]. As a nutritional assessment method, BIA has been validated in patients with CKD and dialysis [55]. It is also an effective tool for early diagnosis of malnutrition in dialysis patients [18].

Other nutritional assessment methods that can be used for dialysis patients include renal inpatient screening tools (renal iNUTs) [56,57]. The application area of this method is limited; hence, it has not been widely used.

Many studies suggest that appropriate nutrition assessment methods should be selected for identification and management of malnutrition in dialysis patients at the earliest as it is important to improve the survival rate and quality of life of dialysis patients.

## 4. Risk factors of malnutrition in dialysis patients

**Figure 1.**
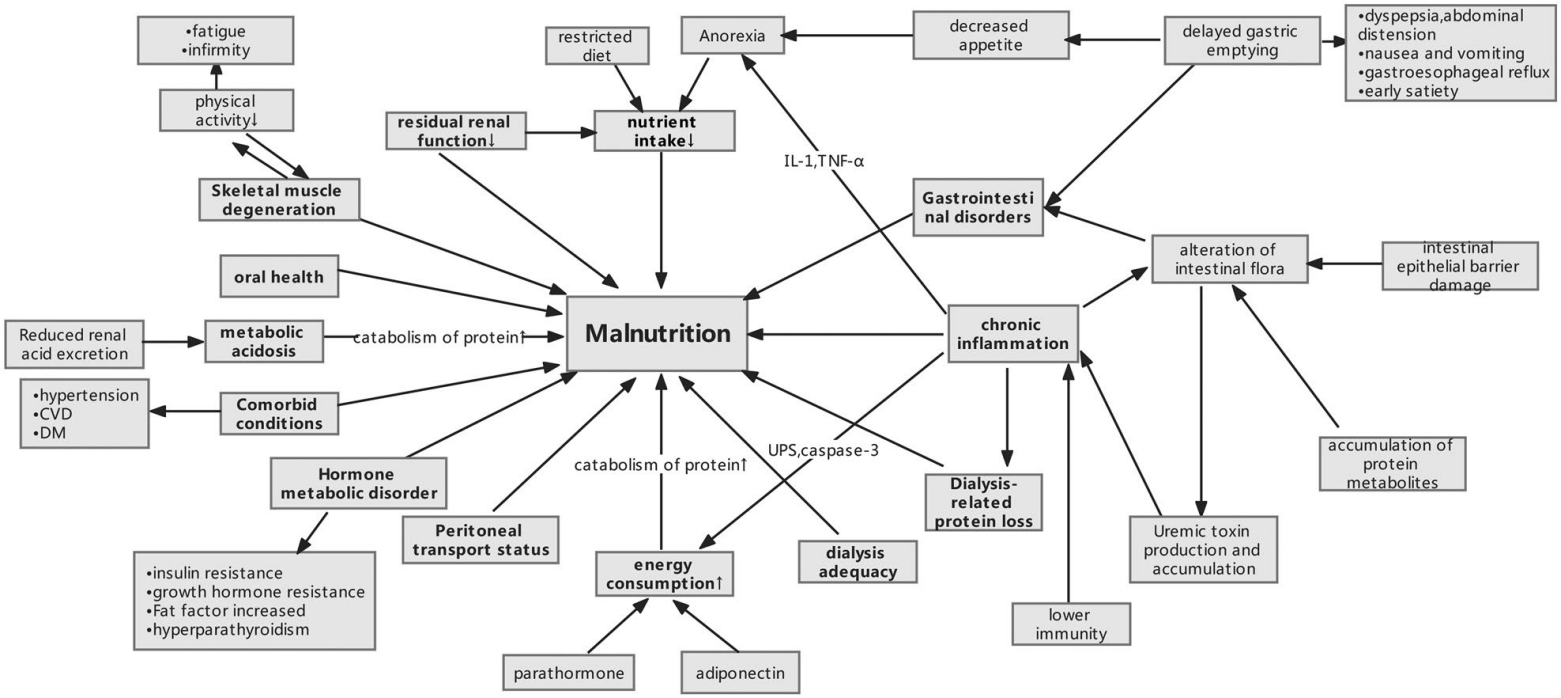
Schematic diagram of malnutrition risk factors and consequences. Abbreviations: DM: diabetes mellitus; CVD: cardiovascular disease; UPS: ubiquitin-proteasome system.

Risk factors of malnutrition in dialysis patients are numerous and interact with each other (Figure 1). Factors such as reduced nutrient intake, microinflammatory state, metabolic acidosis, increased energy expenditure, and endocrine and gastrointestinal disorders will be addressed one by one in the following text.

### 4.1. Reduced nutrient intake

A historical cohort study including 2221 maintenance hemodialysis patients [58] showed a gradual increase in their BMI >20 kg/m^2^ with increase in protein catabolic rate (nPCR), demonstrating that reduced nutrient intake contributes largely to malnutrition in dialysis patients.

Anorexia nervosa and dietary restrictions are the main reasons for experience of reduced nutrient intake among dialysis patients [58,59]. Anorexia nervosa is common in dialysis patients, with a prevalence of 29.5–50% [60–62]. According to many previous studies, it has been found that the causes of anorexia may be related to central loss of appetite caused by toxins of uremic substances, chronic inflammation, changes in hormones affecting appetite, accumulation of metabolic wastes in the body, and abnormalities in taste buds or taste sensation due to long-term application of oral medications [10,59,63], which leads to a reduced intake of active nutrients in dialysis patients with ESRD. Gołębiewska et al. showed [64] that after 3 months of treatment with megestrol (a synthetic progestin that has been shown to increase appetite in patients [65]), the weight and BMI of dialysis patients increased significantly compared to the previous period and the changes were statistically significant (*p* < 0.05); a decreasing trend towards total cholesterol concentration in the first 2 months of treatment (*p* = 0.052) was also seen. This suggests that as the appetite of dialysis patients improves, so does their nutritional status.

In addition to this, in order to prevent and correct some metabolic complications and delay progression of the disease, dialysis patients are usually recommended to restrict the intake of certain nutrients, such as protein, potassium, sodium, and phosphorus [1,66,67]. Although restriction of protein intake is relatively reduced after initiation of regular dialysis, the intake of potassium, sodium, and phosphorus is still quite restricted, which means that patients can consume a reduced variety of foods, such as some sodium-rich condiments, fruits, vegetables, animal offal, and other crops that are rich in potassium and phosphorus. Although strict sodium restriction can effectively reduce the patient’s water and sodium retention and slow down edema, sodium restriction also makes the dialysis patient’s diet lighter, which undoubtedly aggravates the patient’s loss of appetite and further reduces the intake of nutrients.

In addition to anorexia and dietary restrictions, advanced age, loss of residual renal function, gastrointestinal dysfunction, depression, poor socioeconomic status, and early satiety in peritoneal dialysis may cause reduced nutrient intake in dialysis patients [68–70].

### 4.2. Increased energy consumption

In addition to reduced nutrient intake, increased energy expenditure also plays an important role in malnutrition of dialysis patients. A study by Ikizler et al. [71] reported that resting energy expenditure on non-dialysis days was significantly higher in dialysis patients than in healthy controls and that resting energy expenditure increased further on dialysis days. Resting energy expenditure during dialysis is increased by approximately 15–20% [71]. Increased resting energy expenditure accelerates the consumption of fat and muscle tissue and promotes the catabolism of fat and protein.

The key to increased resting energy expenditure is transformation of white fat to brown fat, a phenomenon known as adipose browning [72]. Mitochondrial uncoupling protein 1 (UCP1) uncouples mitochondrial respiration to generate more heat and brown adipose tissue, leading to lipid mobilization and energy consumption [73]. An animal experiment by Cheung et al. also showed [74] that the activity of UCP1 is increased in mice after nephrectomy, which increases the metabolic rate. It was also found that the mice after nephrectomy had decreased appetite and body weight, and even after force-feeding, the mice did not show a significant increase in their body weight and fat mass. This suggests that the kidneys play a role in regulating metabolism.

Hyperparathyroidism is common in end-stage dialysis patients. It has been found that parathyroid hormone plays a key role in the browning of adipose tissue and increased resting energy expenditure in dialysis patients. Cuppari et al. [75] measured resting energy expenditure in dialysis patients with hyperparathyroidism and found that parathyroid hormone was an independent determinant of resting energy expenditure. And the researcher also found that 6 months after surgery in patients with severe hyperparathyroidism, parathyroid hormone levels and the patients’ resting energy expenditure were significantly reduced. Regarding the mechanism by which parathyroid hormone increases resting energy expenditure and adipose tissue browning, Kir et al. [76] found that parathyroid hormone and parathyroid hormone-related protein (PTHrP) in dialysis patients can increase the expression of thermogenic genes, accelerate adipose tissue browning, and increase resting energy expenditure in dialysis patients. In 2022, a retrospective study by Disthabanchong et al. [77] showed that patients with severe hyperparathyroidism had a poorer nutritional status than dialysis patients with normal or moderate hyperparathyroidism.

Additionally, dialysis patients are generally in a chronic inflammatory state and these inflammatory factors can act on the central nervous system to reduce appetite and increase resting energy expenditure of patients [78].

### 4.3. Metabolic acidosis

Metabolic acidosis is also prevalent in dialysis patients due to decreased ability of the kidneys to excrete acid in patients with ESRD. A higher pH may be more conducive to protein synthesis and may improve the patient’s malnutrition [79,80]. In 2009, in a cell culture study by Chiu et al. [81], it was found that the rate of intracellular protein synthesis in cell cultures increased progressively with increasing pH of the culture medium. In the same year, Mehrotra et al. [82] also found that a significant increase in net positive nitrogen balance was observed when the arterial pH was increased from 7.37 to 7.44 in patients with peritoneal dialysis. In a randomized controlled trial including 134 patients with stage 4 CKD [83], it was found that an increase in serum bicarbonate levels to 24 mmol/L compared to maintaining these levels at 20 mmol/L showed a significant improvement in mid-arm muscle circumference and serum albumin of patients and also delayed the progression of CKD.

Why does acidosis accelerate protein catabolism and exacerbate malnutrition in CKD patients? The mechanism of this was shown in a study by Bailey et al. [84] in 1996, who suggested that metabolic acidosis exacerbates malnutrition in CKD patients by activating the ubiquitin-proteasome system (UPS) to increase protein catabolism. A recent study [85] shed new light on the mechanism and stated that cysteine aspartate protease-3 (Caspase-3) also plays a role. The researchers found that caspase-3 cleaves myosin and myogenic fibers, providing a suitable substrate for UPS-mediated proteolysis. In addition, caspase-3 can also activate 26S proteinosome-mediated protein decomposition by cleaving subunits of 19S proteinosome particles (Rpt2 and 6). Therefore, these tests reveal that the presence or absence of acidosis is closely related to good or bad nutritional status of the patient, and that correction of acidosis in the patient is beneficial for improvement of his nutritional status.

### 4.4. Chronic inflammatory state

CKD is a chronic wasting disease; patients on long-term dialysis therapy are commonly immunocompromised and prone to infection. Therefore, chronic inflammatory states are prevalent in this population. In addition to the common inflammatory cytokines such as interleukin (IL)-6, IL-1β, IL-18, tumor necrosis factor (TNFα), IL-8, and C-reactive protein (CRP), hypoalbuminemia and elevated ferritin levels are also considered as inflammatory markers in patients with ESRD [86–89]. Inflammation-induced malnutrition is achieved through increased proteolytic metabolism and/or decreased protein intake [90]. Inflammatory cytokines can promote protein catabolism by inhibiting phosphatidylinositol 3-kinase (PI3K) activity, which in turn activates two proteolytic pathways, the ubiquitin-proteasome proteolytic system (UPP) and caspase-3, causing a negative nitrogen balance in patients, which in turn leads to malnutrition in dialysis patients [91]. As for reduced protein intake, the inflammatory factor IL-1 can cause anorexia by directly affecting the satiety center, suppressing the patient’s appetite, resulting in reduced protein intake, and malnutrition [92].

### 4.5. Disorders of hormone metabolism

Kidneys have a very important endocrine function and play an important role in the synthesis, metabolism, and regulation of many hormones. In patients with CKD, the ability of the kidneys to regulate various hormones is gradually reduced due to the deteriorating kidney function. Therefore, disorders of hormone metabolism are very common in patients with CKD, especially in those with ESRD.

In 1981, DeFronzo et al. [93] found that insulin resistance occurs early in the course of CKD and becomes more pronounced as renal function continues to deteriorate. There is a strong link between insulin resistance and increased protein catabolism and muscle atrophy [94,95]. Insulin resistance activates the UPP and increases caspase-3 activity, which promotes protein catabolism and causes muscle atrophy [96,97].

In addition to insulin resistance, growth hormone resistance also occurs in patients with ESRD [98,99]. In a study on pediatric patients with CKD [100], the use of recombinant growth hormone (rhGH) was reported to promote growth among these patients. This finding may also demonstrate that growth hormone resistance plays a role in the development of malnutrition in patients with ESRD.

In addition to disturbances in insulin and growth hormone metabolism, elevated adipokines such as leptin, adiponectin, and endolipin can be found in patients with ESRD. Adipocytokines such as adiponectin and leptin are important nutrients for dialysis patients. Coimbra et al. showed that [101] increased adiponectin in chronic dialysis patients induces a more protective high-density lipoprotein profile for the human body, and adiponectin may also have the effect of improving common oxidative stress in hemodialysis patients [102]. Studies [101,103,104] have also shown that higher leptin levels are associated with higher BMI and adipose tissue mass. Unlike healthy individuals, dialysis patients with obesity or higher BMI have better nutritional status and clinical outcomes [105]. However, leptin is a hormone that can regulate appetite [106]. High levels of leptin suppress appetite in patients with ESRD and can lead to increased energy expenditure [107–109]. Although adiponectin has anti-inflammatory, anti-atherosclerosis, and insulin sensitization effects, adiponectin can also increase the energy consumption of patients [110,111], thereby accelerating body’s catabolism and causing malnutrition. There is a significant positive correlation between serum lipocalin levels and malnutrition (*p* < 0.0001) [112]. Elevated levels of endolipin in dialysis patients suppress appetite and reduce serum amino acid levels [113].

In addition, disorders of parathyroid hormone metabolism are also prevalent in dialysis patients, and the mechanisms by which parathyroid hormones cause malnutrition in dialysis patients have been described previously and will not be repeated here. Therefore, it is important to pay attention to and make efforts to correct hormone metabolism disorders in patients with ESRD for the prevention and treatment of malnutrition.

### 4.6. Gastrointestinal disorders

Two major features of gastrointestinal dysfunction in patients with CKD are dysbiosis of the intestinal flora [114–116] and delayed gastric emptying [117]. Dysbiosis of the intestinal flora is caused by exacerbation of the chronic inflammatory state of dialysis patients, which in turn leads to malnutrition [63]. According to the preferential metabolic pathways, the human intestinal flora can be divided into two categories, namely, preferentially fermented carbohydrates and preferentially fermented proteins. Among them, the intestinal bacteria that preferentially ferment proteins produce some potentially toxic substances while decomposing proteins. In healthy people, these harmful substances can be discharged *in vitro* through the kidney; however, in case of renal failure, these harmful substances accumulate in the body, thereby further aggravating the intestinal flora imbalance. In addition to this, re-hydrolysis of urea accumulated in the patient’s intestine by microorganisms produces large amounts of ammonia; this can be further converted into ammonium hydroxide, which damages the integrity of the intestinal epithelial barrier, increases permeability of the intestine, and promotes transfer of toxic substances from the intestine to the circulatory system [118]. Therefore, dysbiosis of the intestinal flora in patients with ESRD promotes the production and accumulation of uremic toxins, while disruption of the intestinal epithelial barrier promotes the absorption of uremic toxins; these absorbed uremic toxins induce or even aggravate the inflammatory state of dialysis patients and makes them malnourished [119]. A recent randomized controlled study [120] has confirmed that supplementation of dialysis patients with enteric probiotics for 2 months significantly increases the patients’ albumin levels, upper arm circumference, and triceps skinfold thickness, improves the patients’ malnutrition status, and decreases the patients’ inflammatory factor levels.

Delayed gastric emptying (gastroparesis) is also very common in patients with ESRD [117,121]. Due to long-term chronic delayed gastric emptying, patients experience gastrointestinal symptoms such as dyspepsia, bloating, nausea and vomiting, gastroesophageal reflux, and early satiety; these gastrointestinal symptoms reduce the patient’s appetite, resulting in decreased nutrient intake and progressive malnutrition [122–124]. When patients improve delayed gastric emptying with the application of pro-gastric motility drugs, their nutritional status improves along with it [125]. In patients on peritoneal dialysis, high retention of peritoneal fluid (which can increase intra-abdominal pressure) and reabsorption of glucose from the peritoneal fluid can cause delayed gastric emptying [126,127]. Other factors affecting delayed gastric emptying include diabetes mellitus and abnormal gastric electromyographic activity [128]. In conclusion, aggressive treatment of gastrointestinal dysfunction not only leads to improvement of malnutrition in patients, but also delays the progression of renal disease.

### 4.7. Skeletal muscle degeneration

Patients on long-term dialysis treatment often experience fatigue due to various physical and psychosocial factors, which directly leads to less physical activity [129,130]. Prolonged reduction in physical activity decreases the muscle mass and strength of patients [131], which in turn leads to degeneration of skeletal muscles, while degeneration of skeletal muscles further reduces physical activity and aggravates malnutrition in patients. It is important to provide appropriate physical exercise to ESRD patients for prevention of skeletal muscle degeneration [132,133]. In a study, Johansen showed that appropriate resistance exercise not only improves physical function in daily life among patients with ESRD, but also has a direct effect on reducing muscle wastage in patients [134]. A recent study [135] revealed that dialysis patients show a significant decrease in the levels of inflammatory markers after 6 months of resistance exercise training. The study also showed significant improvements in serum albumin levels, muscle mass, and physical performance in patients after exercise training. Therefore, increasing resistance exercise training in dialysis patients may reduce muscle atrophy, prevent skeletal muscle degeneration, improve the nutritional status of patients, and delay progression of the disease.

### 4.8. Dialysis-related protein loss

Dialysis can discharge various harmful substances and excess metabolic wastes from the body of patients with ESRD, correct water and electrolyte acid-base balance disorders, and facilitate blood purification. Although the current dialysis technology is quite sophisticated, many complications directly related to dialysis still prevail, one of which is protein and nutrient loss from the body [136–138].

For patients treated with peritoneal dialysis, the most commonly used peritoneal dialysis solution in clinical practice is glucose as the permeate. A single 1.5–4.25% instillation of peritoneal dialysis fluid can have a caloric load of 50–300 kcal, which is equivalent to 0.30% of the total daily energy intake of patients on peritoneal dialysis [21]. Although absorption of glucose in the peritoneal dialysis solution does not reduce the patient’s caloric and protein intake, it is still believed that these ‘calories’ in the peritoneal dialysis solution have an impact on the patient’s protein and other nutrient intake. In addition to caloric absorption of peritoneal dialysis fluid, there is also a significant loss of protein during the process of peritoneal dialysis. Patients treated with peritoneal dialysis lose 6–8 g of albumin-based protein per day with the effluence of peritoneal dialysis fluid, and protein loss in this manner is further increased during peritonitis [139,140].

As with peritoneal dialysis, hemodialysis can also lead to loss of protein and other nutrients. Related studies [137,141,142] have shown that approximately 6–12 g of amino acids and 7–8 g of protein are lost during each dialysis session in patients and these lost proteins are likely to lead to hypoalbuminemia and aggravation of malnutrition in patients.

In addition to the direct loss of protein caused by dialysis itself, Ikizler [143] et al. clearly demonstrated that hemodialysis also increases protein catabolism, by measuring protein synthesis and catabolism in hemodialysis patients.

In addition to the risk factors mentioned above, loss of residual renal function, peritoneal transit status, dialysis adequacy, oral health of the elderly, and comorbidities also have an impact on the nutritional status of dialysis patients, and further studies are needed on the topic [12,19,21,144,145].

## 5. Advances in research on trace elements and malnutrition

Numerous studies have shown that [146–148] the imbalance of trace elements, such as Zinc (Zn), Se, Copper (Cu), etc., in dialysis patients is very common. These trace elements are not only involved in oxidative stress and anti-inflammatory processes in patients, but their imbalance can also lead to malnutrition in dialysis patients.

Zn is an essential trace element in the human body, which has anti-inflammatory and anti-oxidant effects [149,150]. Zn deficiency is common in dialysis patients [148,151]. In 2009, Sahin et al. [152] proposed that low Zn levels in dialysis patients may cause malnutrition, and pointed out that dialysis patients need Zn supplementation to prevent progressive malnutrition. Subsequent studies have also confirmed that [153–158] lower Zn levels are independently associated with higher nutritional risk in dialysis patients. After receiving Zn supplementation, serum Zn levels and oxidative stress state improve in dialysis patients. The patient’s hemoglobin and albumin levels also increase significantly, thereby, effectively improving the nutritional status of dialysis patients. Fukasawa et al. [159] evaluated the nutritional status of dialysis patients by measuring abdominal fat mass and found that there was a significant positive correlation between serum Zn level and abdominal fat area in dialysis patients, and Zn level was an independent predictor of visceral fat area. Additionally, Zn affects the secretion of leptin [160]. Zn deficiency increases the level of leptin in patients [161], which affects the appetite of patients, and may lead to anorexia [162]. Zn supplementation can improve taste abnormalities of dialysis patients [163], and increase dietary intake and weight of patients [164]. Gao et al. [165] showed that Zn supplementation can reduce oxidative stress in peritoneal mesothelial cells (PMcs) to inhibit the effect of high glucose (HG) on epithelial-mesenchymal transition (EMT), thereby, improving peritoneal fibrosis (PF) during peritoneal dialysis. Zn deficiency is a predictor of cardiovascular death in dialysis patients [166], and decrease of Zn levels in dialysis patients is significantly correlated with diastolic dysfunction [167]. Latest research [168–171] states that zinc supplementation also has a positive effect on the patients’ renal function, and low Zn levels in dialysis patients are independent predictors of mortality and infection-related hospitalization.

Trace element Se is a key component of various enzymes in the body and plays an important role in reducing oxidative stress and inflammation in dialysis patients [172]. Se deficiency is common in dialysis patients [146,173,174]. In the 1990s, it was reported that there is a certain correlation between Se and serum albumin levels in dialysis patients [175,176]. In 2011, a study [177] demonstrated that serum Se concentrations were significantly lower in the hemodialysis group than in the control group (30 healthy volunteers) (*p* < 0.01), and that serum Se levels in patients in the hemodialysis group were significantly and positively correlated with their nutrition-related indicators such as albumin and high-density lipoprotein-cholesterol (HDL-C). The trial concluded that the low blood Se status in hemodialysis patients may be associated with malnutrition in patients. In 2012, Yang et al. [170] recruited 111 patients on maintenance hemodialysis, measured serum levels of Se, Cu, and Zn and followed them up for two years; they found that patients with lower serum Se (*p* = 0.026) and Zn (*p* = 0.001) levels were more likely to be hospitalized for infectious diseases. In 2013, a randomized double-blind placebo trial by Salehi et al. [178] found that treatment of hemodialysis patients with Se supplementation for 12 weeks significantly lowered the SGA scores and MIS in the Se group compared to the placebo group (*p* < 0.001), in addition to a significant decrease in IL-6 levels in the Se supplementation group (*p* = 0.016). Thereafter, in a prospective longitudinal study including 1278 hemodialysis patients [179], blood concentrations of 25 trace elements were assessed and followed over time, showing that low blood Se levels in patients were significantly associated with their all-cause hospitalization and mortality rates. In 2021, a cross-sectional study by Liu et al. including 118 patients on hemodialysis treatment used the 2002 Nutritional Risk Screen (NRS 2002) to assess the nutritional status of patients and showed that lower blood Se levels were independently associated with high nutritional risk in maintenance hemodialysis patients [153]. A recent report on peritoneal dialysis patients reported that 41.4% of 406 peritoneal dialysis patients had Se deficiency (< 0.8umol/L), and lower Se levels were associated with reduced dietary intake and increased weakness and inflammation [174]. In addition, Se deficiency is also associated with the risk of all-cause mortality in dialysis patients [180], severe sleep disorders [181], and low response to erythropoietin (ESA) [182].

Cu is also a basic trace element. It is a component of various Cu enzymes in the human body and participates in various physiological processes in the body. However, excessive blood Cu levels can induce oxidative stress in the body, leading to lipid peroxidation and protein oxidation [183]. Compared with healthy people, the level of blood Cu in dialysis patients was significantly increased [184,185]. There was a significant correlation between high blood Cu levels and dyslipidemia, inflammation, cardiovascular disease, and high risk of death in dialysis patients [179,185,186]. As Cu/Zn ratios are increasingly recognized as biomarkers of inflammation [187], a greater number of studies [153,188] have shown that higher Cu/Zn ratios are independently associated with nutritional risk in dialysis patients. Moreover, a recent cross-sectional study by Zuo et al. [189] reported that high Cu/Zn ratio was independently associated with anemia in dialysis patients. Although Zn supplementation can reduce the Cu/Zn ratio and improve anemia in dialysis patients, Takahashi’s results suggest that inappropriate Zn supplementation can lead to Cu deficiency, which directly affects the role of ceruloplasmin and lysine oxidase [190]. Therefore, blood Cu levels should be monitored to avoid Cu deficiency during Zn supplementation in dialysis patients [191].

In addition to the above three trace elements, the imbalance of other trace elements also affects the nutritional status of dialysis patients. Appropriate levels of chromium (Cr) can reduce oxidative stress and inflammation; however, very high levels of Cr have a negative impact on the human body. Cr can accumulate in the bones of patients with ESRD and blood Cr levels are significantly elevated in dialysis patients [192]. Hsu et al. [193] investigated the blood Cr level and nutritional status of 647 hemodialysis patients and found that blood Cr level was significantly negatively correlated with malnutrition. Manganese (Mn) is a cofactor of manganese superoxide dismutase (MnSOD) enzyme. Its deficiency can decrease MnSOD activity and aggravate oxidative damage in human body [194,195]. The level of blood Mn in dialysis patients decreased, and the level of blood Mn was independently correlated with the level of hemoglobin [196,197]. For iron deficiency in dialysis patients [198], many studies have been conducted and their results are relatively clear; hence, we will not repeat them here.

Therefore, in order to monitor and prevent malnutrition in dialysis patients, we should pay attention to the intake of trace elements and their assessment.

## 6. Potential mechanisms by which Se may improve malnutrition

Patients with ESRD treated by dialysis have significantly lower blood Se levels than the healthy population due to decreased appetite, inadequate dietary intake, and its loss during dialysis [199]. Se is an important trace element in the human body, which has a regulatory role in maintenance of metabolic activities of the body [200]. In the body, it is involved in various physiological processes in the form of selenoproteins containing selenocysteine in the active center [201,202].

**Figure 2.**
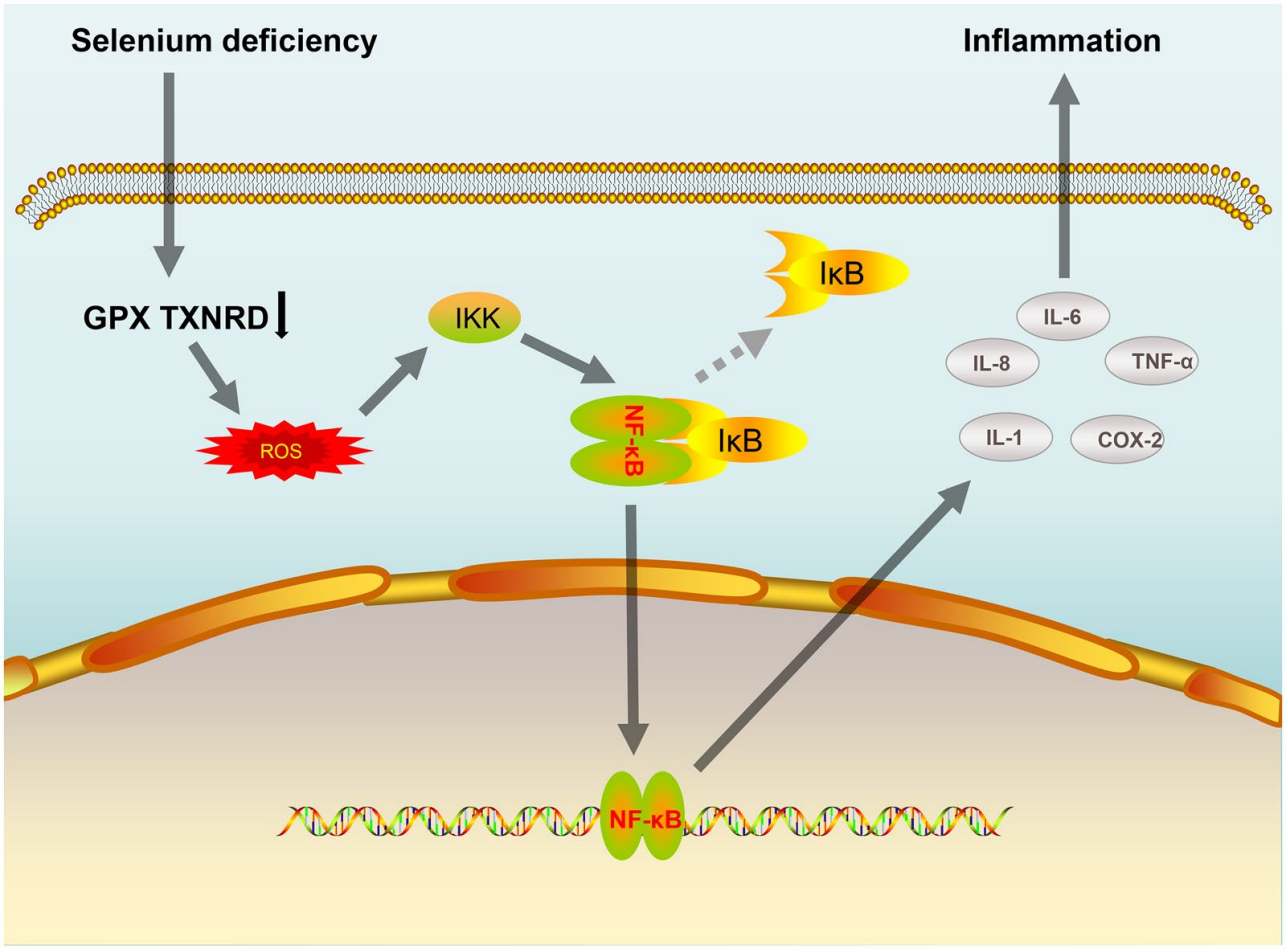
A simple mechanism by which Se deficiency induces inflammation by activation of the NF-κB pathway. Abbreviations: Se: Selenium; GPX: glutathione peroxidases; TXNRD: thioredoxin reductase; ROS: Reactive oxygen species; IKK: IκB kinase; IκB: inhibitor of nuclear factor kappa B; NF-kB: nuclear factor kappa B; IL-1: interleukin-1; IL-6: interleukin-6; IL-8: interleukin-8; COX-2: cyclooxygenase-2; TNF-α: tumor necrosis factor alpha.

Till date, 25 selenoproteins have been identified in humans, including glutathione peroxidases (GPx1-GPx4 and GPx6), members of the thioredoxin reductase family (TXNRD1-TXNRD3), and methionine sulfoxide reductase (MSR B1) [203–205]. Selenoprotein is a key component of the antioxidant defense system and plays an important role in maintaining redox homeostasis in the body. In contrast, a state of oxidative stress and inflammation is prevalent in patients treated with dialysis. A study by Salehi et al. [178] demonstrated that Se supplementation group significantly reduced IL-6 production by inhibiting the activation of nuclear factor-κB (NF-κB) signaling pathway in dialysis patients and concluded that Se supplementation improves the nutritional status of dialysis patients probably by inhibiting oxidative damage and inflammation. Similarly, in a trial by Stockler et al. [206], it was shown that treatment of dialysis patients with Se supplementation for 3 months led to a significant increase in their GPx activity and 8-hydroxytryptamine, 8-isoprostane, TNF-α and IL-6 levels were significantly decreased. This paper briefly summarizes the mechanism by which Se deficiency induces inflammation by activating the NF-κB pathway, as shown in Figure 2.

In addition to the fact that direct Se supplementation improves oxidative stress and inflammation in dialysis patients, the study by Xu et al. [207] revealed the important role played by Se in oxidative stress and infection. His study showed that the application of Lactobacillus casei ATCC 393 (L. casei 393)-Se nanoparticles (Se-NPs) prevented pathogenic Escherichia coli K88 (ETEC K88)-induced intestinal epithelial barrier (IEB) dysfunction and ameliorated ETEC K88-induced oxidative stress. Se deficiency can also destroy the balance of intestinal flora and cause inflammatory reaction in intestinal tissue cells, leading to intestinal inflammation [208,209].

In addition to its anti-oxidant and anti-inflammatory properties, Se supplementation to improve malnutrition in patients may also be associated with its effects on fat digestion and absorption, nutrient utilization, reduction of ketone bodies, and improved insulin action [210–212]. Therefore, additional pilot studies are needed to further explore the potential mechanisms by which Se may improve malnutrition in patients.

## 7. Summary and prospect

In conclusion, malnutrition is very common in CKD patients, especially in those on dialysis. Paying attention to the nutritional problems of CKD patients and integrating nutritional therapy throughout the treatment of CKD has great significance for improvement in the overall diagnosis and treatment of CKD, delayed disease progression, improved patient prognosis, and reduced medical expenses. Many of these studies have shown that blood Se levels in dialysis patients are significantly lower than those in the healthy population, and that blood Se levels are positively correlated with their nutrition-related indicators, while small sample trials have also shown that Se supplementation can improve the malnutrition status of patients. However, there is still a lack of prospective clinical studies with large samples, multicenter-based, and long duration to further prove this conclusion. There is also a lack of basic studies to describe the potential mechanisms by which Se can improve malnutrition in dialysis patients more comprehensively. Most of the current studies have been conducted in patients on maintenance hemodialysis, and data studying the relationship between nutritional status and blood Se in patients on peritoneal dialysis are currently scarce. Therefore, further research is needed to investigate the evidence of correlation between plasma Se and its nutritional status in patients with different dialysis modalities, to provide new diagnostic ideas for predicting and reducing the occurrence of malnutrition events in patients with ESRD treated with dialysis.
